# Influence of Washing on the Thermal Comfort Parameters of Denim Fabrics with Varying Elastane Content

**DOI:** 10.3390/ma19153264

**Published:** 2026-08-02

**Authors:** Zehra Evrim Kanat, Nilgün Özdil

**Affiliations:** 1 Textile and Fashion Design Department, Faculty of Fine Arts, Çanakkale Onsekiz Mart University, Çanakkale 17100, Türkiye; 2 Textile Engineering Department, Faculty of Engineering, Ege University, İzmir 35100, Türkiye; nilgun.ozdil@ege.edu.tr

**Keywords:** denim fabric, elastane ratio, multiple washes, comfort properties

## Abstract

The enduring popularity of denim in the global apparel market is attributed to its outstanding durability, resistance to repeated laundering, and versatility in adapting to changing fashion trends, making it one of the most widely used fabrics in the textile industry. The incorporation of elastane into denim fabrics enhances their elasticity and improves the wearer’s freedom of movement. In addition to stretch performance, optimizing the thermal comfort of stretch denim fabrics has become an important aspect of advanced textile material development. Unlike previous studies, this research provides a comprehensive evaluation of the combined effects of elastane content and repeated home laundering on the structural and thermal comfort properties of stretch denim fabrics. Five denim fabrics containing different elastane ratios were produced, and their dimensional shrinkage, fabric weight, thickness, fractional cover, thermal resistance, air permeability, and relative water vapor permeability were determined before and after five laundering cycles. Statistical analyses were performed to evaluate the effects of elastane content and laundering. The results demonstrated that laundering induced significant structural changes, including increased dimensional shrinkage, fabric weight, thickness, and fractional cover, which consequently altered the comfort performance of the fabrics. Thermal resistance and relative water vapor permeability increased after laundering, whereas air permeability decreased significantly (*p* < 0.05). Correlation analysis further revealed strong statistically significant relationships between the structural characteristics and the measured comfort properties, confirming that both elastane content and laundering are key factors governing the thermal comfort performance of stretch denim fabrics. These findings provide valuable guidance for optimizing the design and laundering performance of stretch denim fabrics with improved thermal comfort and may also contribute to future studies involving functional and technical applications of denim materials.

## 1. Introduction

Denim has long been one of the most widely accepted apparel fabrics, worn by individuals of different ages and social groups owing to its durability, long service life under repeated laundering, and adaptability to changing fashion trends [1]. In recent years, increasing consumer expectations have shifted the focus of denim development beyond durability and esthetics toward enhanced functional and comfort performance [2].

To satisfy these expectations, manufacturers have adopted various technological developments, particularly the incorporation of stretch yarns into denim structures. The use of elastane-containing yarns has become widespread because they improve garment fit, flexibility, and freedom of movement while maintaining the characteristic appearance of denim fabrics [3,4]. Stretch denim fabrics have therefore become increasingly popular due to their ability to conform to body movements without restricting wearer mobility [5,6].

Comfort has become one of the most important quality attributes influencing consumer acceptance of clothing, making the evaluation of textile comfort performance an essential aspect of textile research and product development [7]. The thermophysiological comfort of clothing depends on the ability of textile materials to regulate the transfer of heat, moisture, and air between the human body and the surrounding environment [8]. Clothing should provide adequate thermal insulation while allowing efficient heat and moisture transfer to maintain physiological comfort under different environmental conditions [9]. Heat is transferred through conduction, convection, and radiation, while moisture transport involves water vapor diffusion, sorption, evaporation, and capillary wicking. The efficiency of these transport mechanisms is largely determined by the structural characteristics of the fabric, including its thickness, porosity, and entrapped air. Consequently, thermal resistance, air permeability, and water vapor permeability are key parameters governing heat and moisture exchange, thereby influencing the wearer’s thermal comfort. A comprehensive understanding of the interactions between fabric structure and these transport processes is therefore essential also for the design and optimization of high-performance textile materials [8,10].

Beyond conventional apparel applications, denim fabrics have also attracted increasing attention as reinforcement materials in polymer composites because of their favorable mechanical performance, durability, and sustainability. Recent studies have demonstrated that denim fabric-reinforced composites exhibit improved mechanical and thermal properties, highlighting the potential of denim as a functional material for advanced engineering and technical textile applications [11,12].

Since woven fabrics possess a porous structure that is strongly influenced by their constructional parameters, laundering-induced structural changes are expected to influence these comfort-related properties throughout the service life of denim garments [2].

The comfort performance of denim fabrics is affected by fiber composition, fabric structure, and finishing processes [13]. Previous studies have demonstrated that thermal comfort is strongly influenced by fiber type, yarn structure, fabric thickness, and finishing treatments [14,15]. Mangat et al. reported that both weft yarn material and washing treatments significantly affected the moisture management behavior of denim fabrics [16], whereas Hes and Mangat identified industrial laundering as an important factor governing thermal comfort characteristics [17]. Midha et al. found that unwashed denim fabrics containing cotton weft yarns exhibited significantly higher air permeability and water vapor permeability than fabrics produced with polyester- or elastane-containing weft yarns [2]. Similarly, Güneşoğlu evaluated the influence of hydrophilic polyurethane coating on the physical and comfort-related properties of denim fabrics [18].

Numerous studies have also focused on the influence of elastane on denim performance. Babaarslan et al. investigated the mechanical and comfort properties of denim fabrics produced with multifilament polyester yarns of different filament counts [19], while Meriç and Gürarda evaluated the mechanical properties of elastane-containing woven fabrics [20]. Özdil demonstrated that elastane ratio significantly affected the stretch and bagging behavior of denim fabrics [6]. Eryürük reported that incorporating elastane reduced thermal conductivity, thermal absorptivity, and thermal diffusivity while increasing thermal resistance and water vapor resistance, thereby improving the warm feeling of denim fabrics [4]. Mesfin and Sampath showed that elastane content significantly influenced tensile strength, tear strength, air permeability, stretch, pilling, and drape properties [5]. Likewise, elastane-core and PET/elastane dual-core yarn structures have been reported to reduce air permeability because of their more compact structure [3,21,22]. Okur and Sarıçam demonstrated that hemp incorporation improved moisture management, air permeability, water absorbency, wicking behavior, relative water vapor permeability, drying efficiency, and thermal absorptivity in stretch denim fabrics [23]. Furthermore, Sarker and Mezarcıöz reported that repeated domestic laundering reduced air permeability, tear strength, and stiffness without significantly affecting drying behavior [24], whereas Hosen et al. investigated the influence of different softeners on the thermal and comfort properties of stretch denim fabrics [1].

Stretch denim fabrics produced with cotton/elastane core-spun yarns represent engineered hybrid textile structures, in which the combination of natural and elastomeric components provides a balance between mechanical performance and wearer comfort. Understanding the influence of material composition and laundering on the thermal comfort of these hybrid structures is therefore essential for the development of advanced denim materials. Although previous studies have separately examined the effects of elastane content or laundering on denim comfort performance, a comprehensive evaluation of their combined influence on the thermal comfort characteristics of stretch denim fabrics remains limited. Therefore, this study investigates the effects of repeated laundering on the thermal comfort properties of denim fabrics containing different elastane ratios through detailed structural characterization and statistical analysis.

The remainder of this paper is organized as follows. Section 2 describes the materials, experimental procedures, and testing methods employed in the study. Section 3 presents and discusses the effects of elastane content and repeated laundering on the structural and thermal comfort properties of the denim fabrics, together with the statistical analyses. Finally, Section 4 summarizes the main findings and presents the conclusions of the study.

## 2. Materials and Methods

### 2.1. Materials

**Figure 1:**
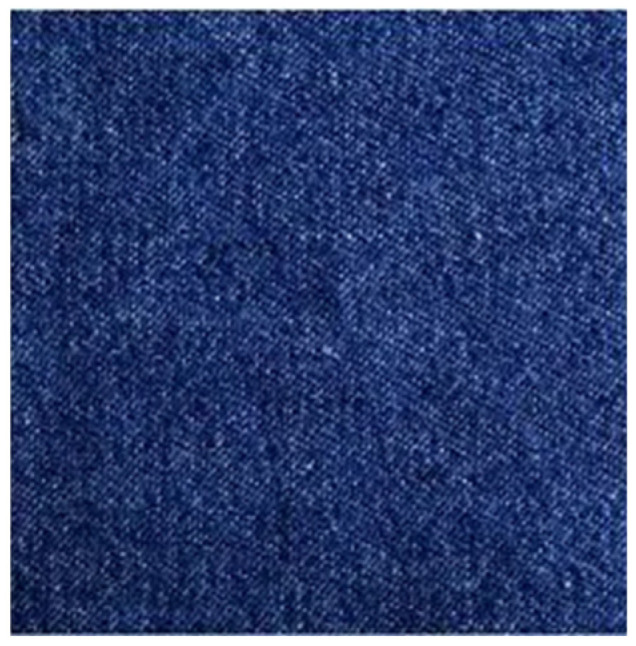
Stretch denim fabric sample.

All fabrics were produced using 100% cotton warp yarns and cotton/elastane core-spun weft yarns. The core-spun weft yarns contained a 78 dtex elastane filament as the core and cotton fibers as the sheath, with an elastane draft ratio of 5.2. The fabrics were woven in a conventional 2/1 Z twill denim construction with a warp density of 27 yarns/cm and a weft density of 20 yarns/cm. A representative image of the fabric is shown in Figure 1**,** and the yarn parameters are presented in Table 1.

The elastane ratios of the denim fabrics were changed with the weft yarn arrangements. These arrangements and the elastane ratios are shown in Table 2.

### 2.2. Method

In the study, fabric tests were performed before and after repeated washing. Thus, the effect of washing on the tested parameters was investigated. Washing procedures were carried out by simulating household conditions. The fabrics were subjected to a total of five washing cycles in accordance with ISO 6330. Washing was performed using a Type A washing machine with 100 g of standard 3-ECE reference detergent, under a 2 kg fabric load, at 30 °C for 40 min per cycle. After washing cycles, the specimens were dried under flat-dry conditions prior to subsequent testing.

Comfort properties of the fabrics before and after washing were compared considering the change in fabric thickness, weight and fractional cover. For this purpose, fabric thickness and fabric weight were determined. Fabric weight was measured according to TS EN 12127. Moreover, the dimensional shrinkage of the fabrics in both the warp and weft directions, after laundering was determined according to TS EN ISO 5077. Also, fractional cover of the fabrics which is the proportion of the fabric surface occupied by warp and weft yarns was calculated using Equation (1) [25].

(1) $$C=(P_{1}\times d_{1}+P_{2}\times d_{2}-d_{1}\times d_{2})/(P_{1}\times P_{2})$$

where

P_1_: distance between two adjacent warp yarns in cm

P_2_: distance between two adjacent weft yarns in cm

d_1_: diameter of warp yarns in cm

d_2_: diameter of weft yarns in cm

**Figure 2:**
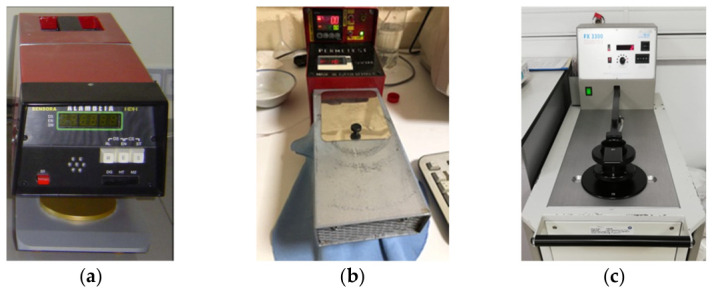
(**a**) Alambeta device (**b**) Permetest instrument (**c**) FX3300 instrument.

Thermal resistance, air permeability, and relative water vapor permeability of the fabrics were evaluated to assess their comfort properties. Thermal resistance and fabric thickness were measured using an Alambeta device (SENSORA, Liberec, Czech Republic), and three measurements were performed for each fabric sample. Air permeability was measured according to TS 391 EN ISO 9237, with seven measurements conducted for each fabric sample using FX3300 instrument (TEXTEST AG, Zürich, Switzerland). Relative water vapor permeability (RWVP) was determined using a Permetest instrument (SENSORA, Liberec, Czech Republic) in accordance with ISO 11092:2014, and three measurements were performed for each fabric sample. The experimental instruments used to evaluate the comfort properties, namely the Alambeta device, the Permetest instrument, and the FX3300 instrument, are shown in Figure 2.

The measurements were performed under standard atmospheric conditions (20 ± 2 °C, 65 ± 4% Rh). The evaluation of the test results was performed using PASW Statistics 18 (SPSS Inc., Chicago, IL, USA).

## 3. Results and Discussion

**Figure 3:**
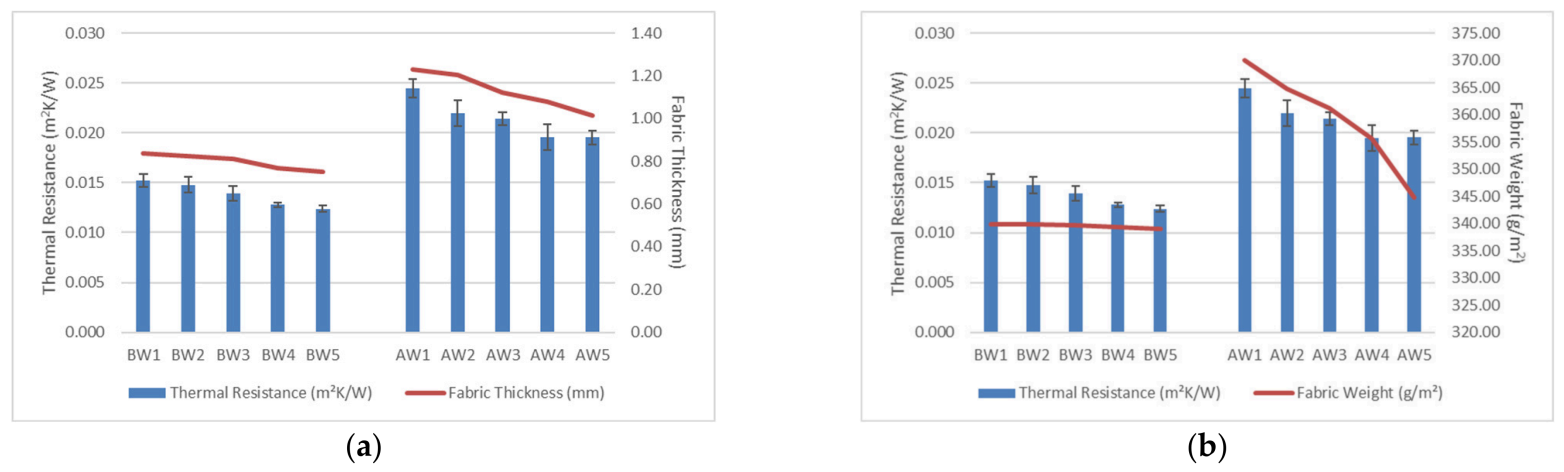
(**a**) thermal resistance vs. fabric thickness before and after washing (**b**) thermal resistance vs. fabric weight before and after washing.

**Figure 4:**
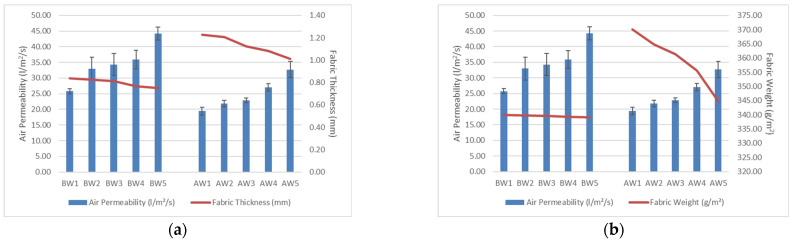
(**a**) air permeability vs. fabric thickness before and after washing (**b**) air permeability vs. fabric weight before and after washing.

**Figure 5:**
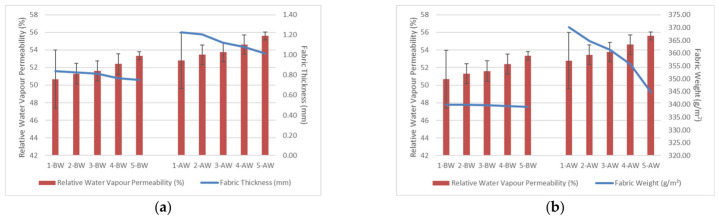
(**a**) relative water vapor permeability vs. fabric thickness before and after washing (**b**) relative water vapour permeability vs. fabric weight before and after washing.

The structural properties of the fabrics, including fabric weight, thickness, and fractional fabric cover, are presented in Table 3. The dimensional shrinkage values of the fabrics in the warp and weft directions after laundering are presented in Table 4. The comfort properties, including thermal resistance, air permeability, and relative water vapor permeability, are presented as arithmetic mean values together with their standard deviations in Table 5. The relationships between the examined parameters with their standard deviations and the fabric’s thickness and weight before and after washing are shown in Figure 3, Figure 4 and Figure 5.

The effect of washing on the thermal resistance, air permeability and RWVP values of the fabrics examined in the study was evaluated with an independent *t*-test (Table 6). In order to determine whether the effects of elastane content in the fabrics on thermal resistance, air permeability, and RWVP values before and after washing were statistically important or not, univariate analysis of variance (ANOVA) was carried out (Table 7).

The dimensional shrinkage results after laundering indicate that the shrinkage values increased with increasing elastane content. In addition, shrinkage was more pronounced in the weft direction than in the warp direction, which can be attributed to the presence of elastane-containing core-spun weft yarns. The higher shrinkage observed in fabrics with greater elastane content resulted in a more compact fabric structure, leading to increases in fabric weight, thickness, and fractional cover. These structural changes are expected to influence the comfort properties of the fabrics by altering heat and moisture transfer as well as air permeability. Therefore, the dimensional changes induced by laundering should be considered together with the structural parameters when evaluating the thermal comfort performance of stretch denim fabrics.

The insulation properties of fabrics are crucial for protection against weather conditions. Since the thermal conductivity of air is very low compared to fibers, a fabric with many small air pockets has higher thermal resistance [14]. As the elastane content in the fabric increases, the fabric thickness increases, thus increasing the thermal resistance. When the effect of washing is evaluated, it can be said that thermal resistance increases depending on thickness and weight (Figure 3a,b). Due to the tightening and increase in weight of the fabrics after the washing process, their thermal resistance has also increased. Statistically, the effect of elastane content in the fabric and washing on thermal resistance was found to be significant (Table 6 and Table 7).

Air permeability is related to the ability of air to penetrate the fabric and unlike thermal resistance, the air permeability of fabrics decreases as fabric thickness and weight increase [14]. Therefore, increasing the elastane content and washing have a negative effect on air permeability (Figure 4a,b). Statistically, it is seen that the effect of washing and elastane content on air permeability is significant (Table 6 and Table 7). In addition, the fractional cover of the fabric increases with the elastane content. Also, an increase in the fractional fabric cover is also observed after washing. The fractional cover value can be defined as the fraction of the areas covered by warp and weft yarns. As this value increases, a decrease in air permeability is expected [2].

Although increases in fabric thickness, fabric weight, and fractional cover were generally associated with a reduction in RWVP in both the unwashed and washed fabrics [14], the increase in RWVP observed after laundering despite the increase in these structural parameters indicates that they alone cannot explain the observed behavior (Figure 5a,b). This phenomenon may be attributed to the structural rearrangement and relaxation of the fabric during washing and drying cycles, which can modify the pore geometry and fabric structure, thereby facilitating water vapor transport. Similar observations have been reported in previous studies [2]. Statistically, while the effect of washing on relative water vapor permeability is significant, the effect of elastane content is insignificant (Table 6 and Table 7).

In order to compare the elastane ratios both before and after washing in terms of thermal resistance and air permeability, variance of homogeneity tests were carried out. Multiple comparisons of the mean values were done according to Tukey’s multiple comparison test when the variances were equal, and according to Tamhane’s multiple comparison test when the variances were not equal.

When examining the multiple comparison tables before and after washing for thermal resistance, it is possible to say that the differences between some fabrics, whose weight and thickness change with washing, decrease (Table 8).

Evaluation of the multiple comparison results for air permeability (Table 9) showed that the elastane content was a statistically significant factor affecting air permeability in both the unwashed and washed fabric groups. The statistical comparisons indicated that the influence of elastane content remained significant after washing. Furthermore, the number of statistically significant differences between certain fabric groups increased after laundering, suggesting that the effect of elastane content on air permeability became more pronounced following the washing and drying cycles.

Correlation coefficient results are shown in Table 10. The results obtained show a high correlation between thermal resistance and washing, fabric thickness and weight, and between air permeability and washing, elastane ratio, fabric thickness and weight. Furthermore, while there is a very strong correlation between relative water vapor permeability and washing, fabric thickness, and weight, the correlation with elastane content is found to be strong.

In the present study, thermal resistance exhibited a very strong positive correlation with washing (r = 0.929), whereas air permeability showed strong negative correlations with washing (r = −0.680) and elastane content (r = −0.691). In addition, relative water vapor permeability was strongly positively correlated with washing (r = 0.753) and negatively correlated with elastane content (r = −0.632). The observed correlations are consistent with previous studies. In a study comparing 100% cotton and 98% cotton/2% elastane denim fabrics, the Pearson correlation coefficient between fabric type and relative water vapor permeability was reported as −0.965 [4]. Similarly, in another study on 100% cotton denim fabrics, fabric weight showed strong negative correlations with relative water vapor permeability (r = −0.936) and air permeability (r = −0.963), while fabric thickness was positively correlated with thermal resistance (r = 0.752) and negatively correlated with relative water vapor permeability (r = −0.646) and air permeability (r = −0.749) [14]. A comparison with the correlation coefficients presented in Table 10 demonstrates that the findings of the present study are in good agreement with the published literature. These findings confirm that both fabric structure and laundering conditions are key factors governing the thermal comfort performance of denim fabrics. Moreover, statistically significant relationships (*p* < 0.05) were observed between the structural parameters and the measured comfort properties, demonstrating the strong influence of fabric structure on the thermal comfort behavior of denim fabrics.

## 4. Conclusions

This research focused on assessing the effects of laundering on the comfort-related properties of denim fabrics with different elastane contents. Five fabric variants were used and exposed to five home washing cycles. The influence of washing on thermal resistance, air permeability, and relative water vapor permeability was investigated through measurements conducted both before and after the laundering process.

The results demonstrated that variations in elastane content significantly influenced thermal resistance and air permeability. Higher elastane ratios were associated with increases in fabric thickness, fabric weight, and fractional cover, which contributed to improved thermal resistance but lower air permeability.

Laundering caused dimensional shrinkage in both the warp and weft directions, with more pronounced shrinkage observed in the weft direction as the elastane content increased. This structural change resulted in increases in fabric thickness, weight, and fractional cover. Consequently, thermal resistance, air permeability, and relative water vapor permeability were altered after laundering, and these changes were found to be statistically significant for all three comfort parameters.

Due to the increase in thickness, weight and fractional cover of the fabrics after washing, thermal resistance increased and air permeability decreased. The swelling of fibers during washing has led to an increase in the RWVP value.

Furthermore, in line with the literature, a very strong correlation was found between measured comfort properties and fabric thickness, weight and fractional cover. In addition, the relationship between elastane ratio and air and water vapor permeability was also very strong.

Washing results in structural changes in the fabrics, increasing their thermal resistance and water vapor permeability while decreasing their air permeability. These changes in comfort properties varied depending on the elastane content. Therefore, to ensure denim fabrics maintain their comfort after washing, the elastane content must be appropriately selected.

The findings of this study also contribute to understanding of the comfort behavior of advanced stretch denim fabrics manufactured with engineered hybrid yarn systems. The results demonstrate that the interaction between material composition, structural characteristics, and laundering conditions plays a decisive role in determining thermal comfort performance. These findings provide useful guidance for the design and optimization of next-generation stretch denim fabrics with improved functional and comfort properties. Moreover, as denim fabrics have increasingly attracted attention as reinforcing materials in composite systems, the present findings may also serve as a valuable reference for future studies and applications where the thermal behavior and structural characteristics of denim materials are important design considerations.

## Figures and Tables

**Table 1:** Yarn parameters.

Parameter	Warp Yarn	Weft Yarn (With and Without Elastane)
Yarn count (Ne)	8	14
Twist coefficient (αe)	4.5	4.2

**Table 2:** Weft Yarn Arrangements.

Fabric Code	Weft Yarn Arrangement	Elastane Ratio (%)
1	All core spun yarns	1.50
2	3 core spun yarns + 1 plain yarn	1.05
3	2 core spun yarns + 1 plain yarn	0.95
4	1 core spun yarn + 1 plain yarn	0.70
5	1 core spun yarn + 2 plain yarns	0.50

**Table 3:** Structural fabric parameters.

Material	Mass per Unit Area (g/m^2^)	Fabric Thickness (mm)	Fractional Fabric Cover
1-BW	339.93	0.84	0.8813
2-BW	339.83	0.83	0.8738
3-BW	339.70	0.81	0.8725
4-BW	339.35	0.77	0.8716
5-BW	339.08	0.75	0.8651
1-AW	370.10	1.23	0.8976
2-AW	364.80	1.20	0.8923
3-AW	361.30	1.12	0.8920
4-AW	355.60	1.08	0.8870
5-AW	344.70	1.01	0.8792

BW—Before washing, AW—After washing.

**Table 4:** Dimensional shrinkage values (%).

Material	Weft Direction	Warp Direction
1	5.50	4.83
2	5.30	3.60
3	5.30	3.50
4	4.60	1.83
5	3.00	1.60

**Table 5:** Mean values and standard deviations of fabric comfort properties.

Material	Thermal Resistance (m^2^K/W)		Air Permeability (L/m^2^/s)		RWVP (%)	
	Arithmetic Mean	Standard Deviation	Arithmetic Mean	Standard Deviation	Arithmetic Mean	Standard Deviation
1-BW	0.0152	0.00066	25.77	0.88	50.67	3.18
2-BW	0.0148	0.00082	33.01	3.66	51.30	1.10
3-BW	0.0139	0.00073	34.30	3.48	51.60	1.10
4-BW	0.0128	0.00025	35.89	2.91	52.40	1.10
5-BW	0.0124	0.00032	44.21	2.11	53.35	0.45
1-AW	0.0245	0.00094	19.41	1.21	52.79	3.31
2-AW	0.0220	0.00130	21.83	1.06	53.45	1.15
3-AW	0.0214	0.00066	22.89	0.73	53.76	1.15
4-AW	0.0195	0.00129	27.03	1.23	54.60	1.15
5-AW	0.0196	0.00069	32.67	2.60	55.59	0.47

BW—Before washing, AW—After washing.

**Table 6:** Independent *t*-test results for washing.

Parameter		N	Mean	St. Deviation	df	t	*p*
Thermal Resistance	Before Washing	15	0.0138	0.00124	28	−12.109	0.000 *
	After Washing	15	0.0214	0.00208			
Air Permeability	Before Washing	35	34.6371	6.54712	68	7.124	0.000 *
	After Washing	35	24.7657	4.93290			
RWVP	Before Washing	15	51.8633	1.70676	28	−3.418	0.002 *
	After Washing	15	54.0400	1.78028			

* Statistically significant according to α = 0.05.

**Table 7:** Variance analysis test results for elastane ratios.

Parameters		df	F	Significance (2-Tailed)
Thermal Resistance	Before Washing	4	12.424	0.001 *
	After Washing	4	12.161	0.001 *
Air Permeability	Before Washing	4	39.013	0.000 *
	After Washing	4	83.217	0.000 *
RWVP	Before Washing	4	1.163	0.383
	After Washing	4	1.164	0.383

* Statistically significant according to α = 0.05.

**Table 8:** Multiple comparisons of thermal resistance among fabric groups using a post hoc (Tukey) for before and after washing.

Group	Groups	Thermal Resistance Before Washing		Thermal Resistance After Washing	
		Mean Difference	Sig.	Mean Difference	Sig.
1	2	0.00041	0.916	0.00252	0.074
	3	0.00130	0.133	0.00309 *	0.025
	4	0.00242 *	0.004	0.00495 *	0.001
	5	0.00281 *	0.001	0.00492 *	0.001
2	3	0.00090	0.411	0.00057	0.954
	4	0.00201 *	0.014	0.00244	0.086
	5	0.00241 *	0.004	0.00240	0.091
3	4	0.00112	0.230	0.00186	0.238
	5	0.00151	0.070	0.00183	0.251
4	5	0.00039	0.924	−0.00003	1.000

* Statistically significant according to α = 0.05.

**Table 9:** Multiple comparisons of air permeability among fabric groups using a post hoc (Tukey) for before and (Tamhane) for after washing.

Group	Groups	Air Permeability Before Washing		Air Permeability After Washing	
		Mean Difference	Sig.	Mean Difference	Sig.
1	2	−7.24286 *	0.000	−2.41429 *	0.020
	3	−8.52857 *	0.000	−3.47143 *	0.001
	4	−10.11429 *	0.000	−7.61429 *	0.000
	5	−18.44286 *	0.000	−13.25714 *	0.000
2	3	−1.28571	0.909	−1.05714	0.427
	4	−2.87143	0.329	−5.20000 *	0.000
	5	−11.20000 *	0.000	−10.84286 *	0.000
3	4	−1.58571	0.825	−4.14286 *	0.000
	5	−9.91429 *	0.000	−9.78571 *	0.000
4	5	−8.32857 *	0.000	−5.64286 *	0.007

* Statistically significant according to α = 0.05.

**Table 10:** Pearson Correlation Coefficients.

Dependent-Independent Variables	Pearson Correlation Coefficients	*p*-Values (2-Tailed)
Thermal Resistance-Washing	0.929	0.000 *
Thermal Resistance-Elastane Ratio	0.346	0.327
Thermal Resistance-Weight	0.940	0.000 *
Thermal Resistance-Thickness	0.989	0.000 *
Thermal Resistance-Fractional Fabric Cover	0.949	0.000 *
Air Permeability-Washing	−0.680	0.030 *
Air Permeability-Elastane Ratio	−0.691	0.027 *
Air Permeability-Weight	−0.826	0.003 *
Air Permeability-Thickness	−0.850	0.002 *
Air Permeability-Fractional Fabric Cover	−0.964	0.000 *
RWVP-Washing	0.753	0.012 *
RWVP-Elastane Ratio	−0.632	0.050 *
RWVP-Weight	−0.888	0.001 *
RWVP-Thickness	−0.913	0.000 *
RWVP-Fractional Fabric Cover	−0.980	0.000 *

* Statistically significant according to α = 0.05.

## Data Availability

The original contributions presented in this study are included in the article. Further inquiries can be directed to the corresponding author.

## Abbreviations

The following abbreviations are used in this manuscript:

BW	Before Washing
AW	After Washing
RWVP	Relative Water Vapor permeability
